# MRI enhances the understanding of critical anatomy during primary laparoscopic port placement

**DOI:** 10.52054/FVVO.15.2.061

**Published:** 2023-06-30

**Authors:** E.A. Layden, R.R. Chodankar, L.E. Kershaw, M Madhra

**Affiliations:** University of Edinburgh, Edinburgh, UK; Royal Infirmary of Edinburgh, NHS Lothian, Edinburgh, UK; Centre for Cardiovascular Science and Edinburgh Imaging, University of Edinburgh, Edinburgh, UK

**Keywords:** laparoscopic surgery, Veress, safety, magnetic resonance imaging, Palmer’s point

## Abstract

Despite the majority of laparoscopic visceral injuries occurring with primary entry, high-fidelity training models are lacking.

Three healthy volunteers underwent non-contrast 3T MRI at Edinburgh Imaging. A direct entry 12mm trocar was filled with water to improve MR visibility, placed on the skin at entry points, then images were acquired in the supine position. Composite images were created, and distances from the trocar tip to the viscera were measured, demonstrating anatomical relationships during laparoscopic entry.

With a BMI of 21 kg/m2, gentle downward pressure during skin incision or trocar entry reduced the distance to the aorta to less than the length of a No. 11 Scalpel blade (22mm). The need for counter-traction and stabilisation of the abdominal wall during incision and entry is demonstrated.

With a BMI of 38 kg/m2, deviating from the vertical angle for trocar insertion can result in the entire trocar shaft being placed within the abdominal wall without entering the peritoneum, creating a ‘failed entry.’

At Palmer’s point distance between the skin and bowel is only 20mm. Ensuring the stomach is not distended will minimise gastric injury risk.

The use of MRI to provide visualisation of the critical anatomy during primary port entry allows the surgeon to gain better understanding of textually described best practice techniques.

## Introduction

The Royal College of Obstetricians and Gynaecologists (RCOG) and British Society of Gynaecology Endoscopy (BSGE) guideline, published in 2008, describes best practice to minimise the risk of injury during laparoscopy (Sutton and Philips, 2008). The characteristic feature of laparoscopic surgery is the insertion of medical devices to gain initial entry into the peritoneal cavity. This initial step is associated with half of all visceral injuries (Harkki-Siren and Kurki, 1997; Jansen et al., 1997). Visceral injury during laparoscopic injury is not always immediately recognised, and this can lead to the development of significant morbidity, such as major abdominal reparative surgery, including colostomy (Sutton and Philips, 2008).

The incidence of entry related major visceral injuries has been reported using various methodologies, across a variety of hospital settings and countries. The overall range of bowel, urological and vascular injuries associated with laparoscopy appears to be between 2.3 and 3.6 per 1000 cases (Querteu et al., 1993; Pierre et al., 1997; Richardson and Sutton, 2001; Jones et al., 2002). In a study where contemporaneous recommended criteria for laparoscopic entry were not met, the rate of bowel injury was reported higher at 4.3 per 1000 cases (Garry, 1999).

There are three established laparoscopic entry techniques: Veress (closed), Hasson (open) and direct trocar entry. The Veress technique involves using the Veress Needle (a needle with a spring- loaded guard) to insufflate the peritoneal cavity with gas prior to the insertion of the primary trocar. The Hasson technique uses a small incision to enter the peritoneal cavity under direct vision, with minimal use of sharp instruments after the initial skin incision (Hasson, 2001). The visualisation of the bowel or omentum confirms that the peritoneal cavity has been entered, and a blunt tipped trocar can be inserted and secured, through which insufflation can occur. Direct trocar insertion was described in 1978 and involves the trocar insertion into the peritoneal cavity without prior insufflation (Dingfelder, 1978). No safety disadvantage with major complications was associated with the latter technique, with a possible reduction in failed entry compared to Veress (Ahmad et al., 2019).

Veress and Hasson’s techniques have reported rates of bowel and vascular injury less than 1 per 1000 (Garry, 2001; Hasson, 2001; Penfield, 1985), with some studies suggesting Hasson has a higher rate of bowel injury (Merlin et al., 2001).

Palmer’s point is 3cm below the left costal margin in the mid-clavicular line and is the recommended site for laparoscopic entry in some cases. Veress or direct entry are typically used here when there is concern about underlying bowel adhesions beneath the umbilicus or at extremes of patient body weight.

Thankfully, bowel and vascular injuries are rare but remain a shared feature across all standard laparoscopic entry techniques. The absence of a definitive superior technique means that the surgeon who aims to keep the risk for their patient as low as possible employs their preferred technique as close to established best practice as possible. There are no established high-fidelity training models for primary laparoscopic entry. The aim of this study is to use MRI to visually demonstrate the rationale behind the best practice and highlight the potential pitfalls of deviating from this.

## Methods

Three healthy volunteers, aged 20 to 35 years with a BMI range of 21 to 39 kg/m2, agreed to undergo a non-contrast 3T MRI at Edinburgh Imaging. An Applied Medical Kii Fios First Entry 12mm trocar was placed at the umbilicus or Palmer’s point, filled with water to improve MR visibility, and images were acquired in a supine position.

Images were acquired with the trocar positioned optimally, in keeping with best practice, and when deliberately positioned sub-optimally, such as oblique placement at 50 degrees from the horizontal or without the effect of stabilisation of the abdominal wall. Composite images were created, and measurements were taken from the tip of the trocar to the potential injury sites. In addition, annotations were created to relate the size of surgical instruments to anatomical structures not visualised during laparoscopic primary port placement.

The assessment of Magnetic Resonance Imaging with healthy volunteers received a favourable ethical opinion from the ACCORD Medical Research Ethics Committee following amendment SA01 on 11th July 2017. All volunteers provided written informed consent before the scan session.

## Results

**Figure 1:**
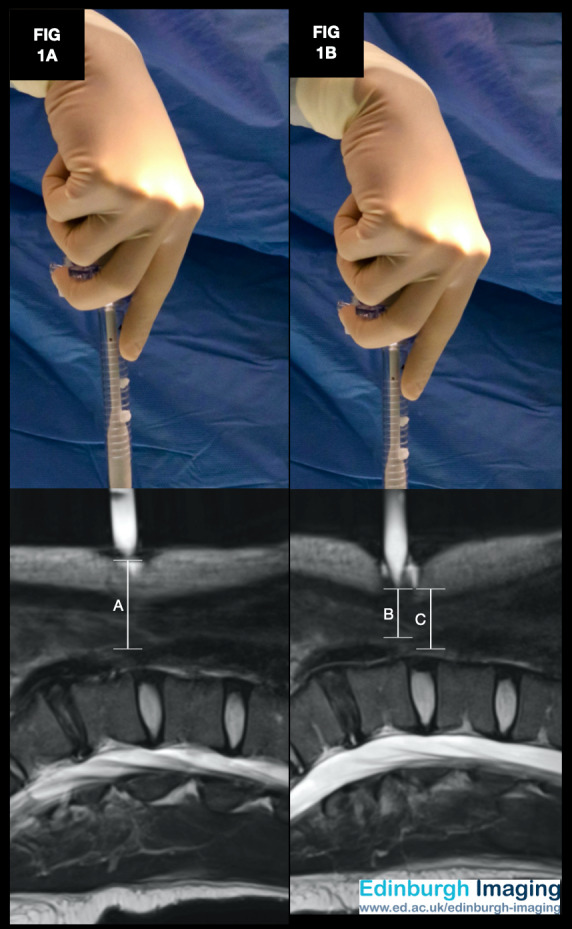
Composite image with MRI in the sagittal plane using a water-filled 12mm direct entry trocar at the umbilicus. This volunteer had a BMI of 21 kg/m2, and with the trocar positioned at rest (Figure 1A), at 90 degrees to the horizontal, at the base of the umbilicus, the distance from the tip to the aorta was 31.9mm (Annotation A). With the same volunteer and trocar position, the addition of gentle downward pressure (Figure 1B) reduced the distance from the trocar tip to the aorta to 17.1mm (Annotation B). The length of an 11 Scalpel Blade (22mm) is marked for comparison (Annotation C).

*Figure1A*: This volunteer had a BMI of 21 kg/m2, and with the trocar positioned at rest, at 90 degrees to the horizontal, at the base of the umbilicus, the distance from the trocar tip to the aorta was 31.9mm. *Figure 1B*: With the same volunteer and trocar position, the addition of gentle downward pressure reduced the distance from to trocar tip to the aorta to 17.1mm.

**Figure 2:**
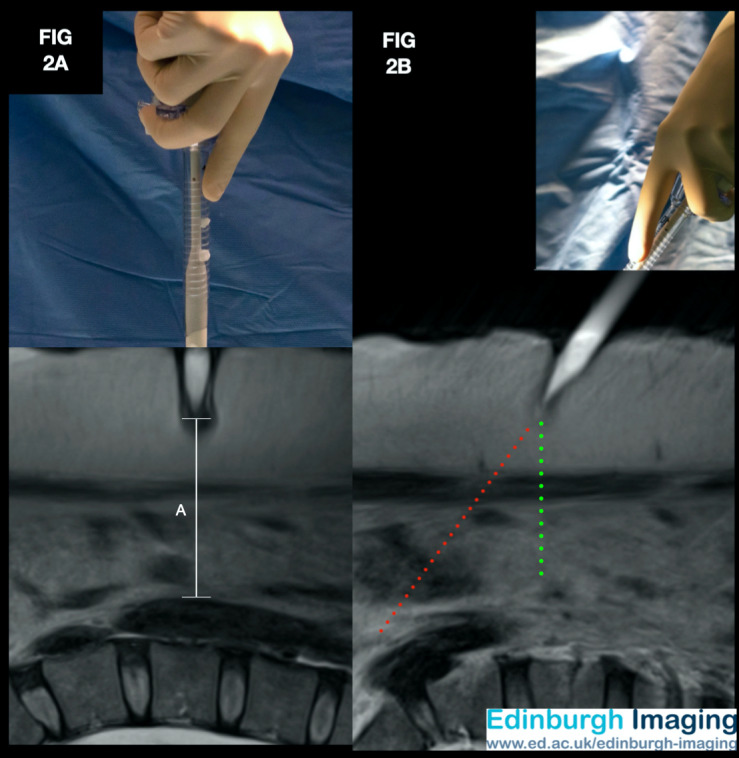
Composite image with MRI in the sagittal plane using a water-filled 12mm direct entry trocar at the umbilicus. This volunteer had a BMI of 39 kg/m2, and with the trocar positioned at rest, at 90 degrees to the horizontal, at the base of the umbilicus, the distance from the tip to the aorta was 67.5mm. (Annotation A) With the same volunteer, the deviation of the trocar shaft to 50 degrees from the horizontal, the distance from the trocar tip to the aorta was over 115mm (oblique dotted line).

*Figure 2A*: This volunteer had a BMI of 39 kg/ m2, and with the trocar positioned at rest, at 90 degrees to the horizontal, at the base of the umbilicus, the distance from the tip to the aorta was 67.5mm. *Figure 2B*: With the same volunteer, the deviation of the trocar shaft to 50 degrees from the horizontal, the distance from the trocar tip to the aorta was over 115mm.

**Figure 3:**
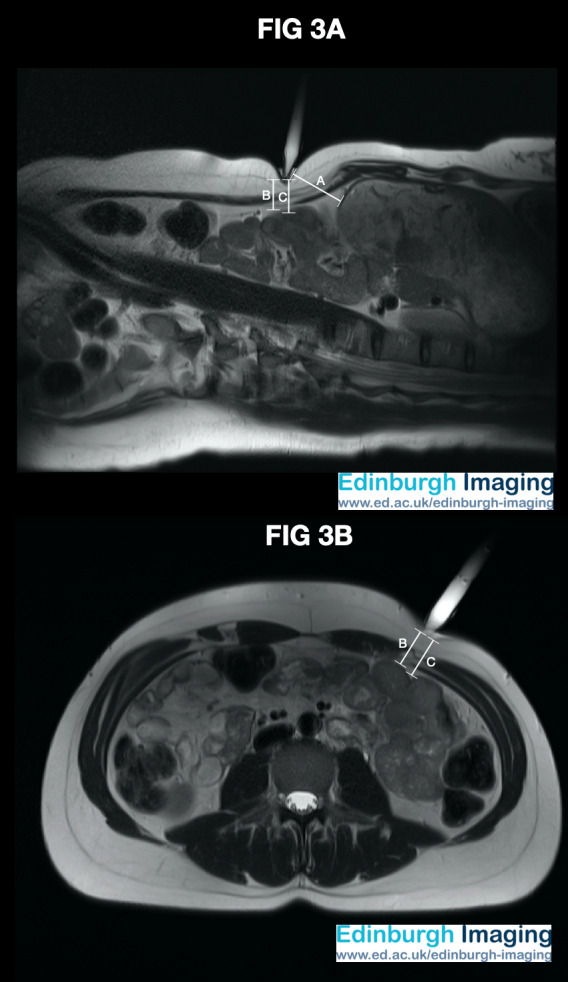
Composite image with MRI in the axial and oblique sagittal plane using a water-filled 12mm direct entry trocar at Palmer’s point. This volunteer had a BMI of 24 kg/m2, and the trocar shaft positioned perpendicular to the skin, the tip at Palmer’s point and gentle downward pressure. The distance to the non-fasted, non-insufflated stomach was 42.6mm (Annotation A). The distance from the trocar tip to the small bowel was 20.0mm (Annotation B). The length of an 11 Scalpel Blade (22mm) is marked for comparison (Annotation C).

*Figure 3A/3B*: This volunteer had a BMI of 24 kg/m2, and with the trocar shaft positioned perpendicular to the skin, the tip at Palmer’s point, and gentle downward pressure, the distance from the trocar tip to the small bowel was 20.0mm. The distance to the non-fasted, non-insufflated stomach was 42.6mm.

## Discussion

There is an established risk of damage to the great vessels for any entry method, but most literature does not describe the mechanism of injury. These images highlight that in order to maintain a safe laparoscopic approach for the patient, anatomy and the relations between structures must be appreciated at all stages of surgery.

Figure 1A/1B demonstrates the distances between the base of the umbilicus and the major vessels, at rest and with gentle downward pressure in a volunteer with a normal range BMI. Figure 1B illustrates the need for stabilisation and counter-traction of the anterior abdominal wall if there is instrument placement without prior pneumoperitoneum creation, such as the Veress or direct trocar entry methods. This image was acquired with gentle downward pressure on the trocar. It is easy to imagine the 17.1 mm distance between the base of the umbilicus and the aorta being reduced further with a greater amount of downward pressure during surgery, risking visceral injury unless appropriate methods are employed to provide counter traction.

The Hasson entry method is also associated with visceral injuries (Merlin et al., 2001). A trainee laparoscopist must remember the location of the sharp tip of the scalpel blade used to incise the skin. Without due care to ensure enough counter- traction to stabilise the abdominal wall during skin incision, retroperitoneal structures such as the aorta or the inferior vena cava could sustain a laceration before the pneumoperitoneum, and laparoscopic vision is established. The length of the scalpel blade is typically greater than the distance to the aorta in patients with a normal or low BMI.

The length of an 11 Scalpel Blade (22mm) is marked for comparison in Figure 1A/1B. Without measures to counter the reduction of the skin to viscera distance, even keeping most of the scalpel blade in direct vision during incision of the base of the umbilicus may be insufficient to avoid harm. A scalpel tip laceration of the great vessels is at risk of being missed during the initial 360-degree survey due to overlying bowel, or any associated haematoma taking time to be of sufficient volume to cause haemodynamic compromise.

Figure 2A/2B demonstrates the entry related problems which can occur by deviating from a perpendicular angle for primary instrument entry in individuals with a raised BMI. A 90-degree angle of engagement between the tip of the trocar at the point of contact with tissue, shown in Figure 2A, means that forces on the tip of the direct entry trocar in the cranio-caudal, and lateral, directions are in balance. This will create a ‘depression’ in the shape of the underlying tissue. The depression allows for the effort from the surgeon to be transmitted to the trocar and be localised at the trocar’s blunt tip. In combination with counter-traction upwards on abdominal wall, an oscillating rotational action will then allow the trocar to puncture the sheath in a safe and controlled manner using the least possible downward force.

When the primary trocar entry is attempted with an oblique angle, as in Figure 2B, there is a mismatch, typically between the cranial and the caudal forces between the trocar tip, and the underlying tissue. Rather than creating a stabilising local environment for safe entry, the effort for the surgeon is instead directed to slide the trocar tip along the surface of the tissue or rectus sheath. An inexperienced surgeon, without considering the sliding effect caused by an imbalance of the forces at the trocar tip, may hope that the oblique dotted line is where the trocar would be projected to enter the sheath. With this belief, onward attempts to enter the sheath will compound the sliding forces at the trocar tip, and the entire length of the trocar can easily be inserted into the abdominal wall or the pre-peritoneal space creating a ‘failed entry.’ The alternative outcome, in this case, is that of an oblique puncture of the sheath by the trocar, more caudal to the umbilicus than intended. There is a risk of this entry being uncontrolled, given the greater overall effort required for the surgeon to puncture the sheath at an oblique angle. Furthermore, using an inferiorly and obliquely positioned primary trocar will restrict the field of view and impair ergonomics for the surgery that follows.

Figure 3A/3B demonstrates a trocar placed at Palmer’s point. The trainee laparoscopist should be guided to place the trocar perpendicular to the skin at Palmer’s point. A vertically, as opposed to perpendicularly, held trocar here would lead to an imbalance of lateral forces at the trocar tip and encourage the tip to slide further to the flank and create a ‘failed entry.’ Additionally, the abdominal wall at Palmer’s point is typically less mobile than at the umbilicus due to its proximity to the costal margin and has less subcutaneous adipose tissue. Without taking these into account, the fully inserted scalpel blade could risk inadvertent damage to the bowel. Figure 3A measured the distance to the non-fasted stomach at 42.6mm. Unintentional insufflation of the stomach during induction of anaesthesia, or a non-fasted patient (for instance, in emergency surgery), can lead to distention of the stomach below Palmer’s point and lead to entry related injury. This risk can be reduced by deflating the stomach with an orogastric or nasogastric tube prior to Palmer’s point entry.

## Conclusions

Laparoscopic surgery, at its core, is a visual endeavour, with sight providing the majority of coordination and feedback to the surgeon. In contrast, the placement of the primary port is based on haptics and requires imagination, with almost no visual aspect. This may be why most visceral injuries occur at the time of laparoscopic entry.

These images create a visual framework to support the tactile and haptic feedback the laparoscopist feels during a primary port entry. In addition, the images of deliberately sub- optimal port placement demonstrate the rationale underpinning best practice techniques to avoid these pitfalls.

Furthermore, the advantages of avoiding unopposed downward pressure on the abdominal wall during all entry techniques are presented. The laparoscopist can use their knowledge of the clinically relevant anatomical relations to refine their approach during primary port entry to make this aspect of surgery as safe as possible for the patient.
